# A Modular Composite Device of Poly(Ethylene Oxide)/Poly(Butylene Terephthalate) (PEOT/PBT) Nanofibers and Gelatin as a Dual Drug Delivery System for Local Therapy of Soft Tissue Tumors

**DOI:** 10.3390/ijms23063239

**Published:** 2022-03-17

**Authors:** Anna Liguori, Alessandro De Vita, Giulia Rossi, Luisa Stella Dolci, Silvia Panzavolta, Chiara Gualandi, Laura Mercatali, Toni Ibrahim, Maria Letizia Focarete

**Affiliations:** 1 Department of Chemistry “Giacomo Ciamician” and INSTM UdR of Bologna, University of Bologna, Via Selmi 2, 40126 Bologna, Italy; giulia.rossi49@studio.unibo.it (G.R.); silvia.panzavolta@unibo.it (S.P.); c.gualandi@unibo.it (C.G.); marialetizia.focarete@unibo.it (M.L.F.); 2 Osteoncology Unit, Bioscience Laboratory, IRCCS Istituto Romagnolo Per Lo Studio Dei Tumori (IRST) “Dino Amadori”, 47014 Meldola, Italy; alessandro.devita@irst.emr.it (A.D.V.); laura.mercatali@irst.emr.it (L.M.); 3 Department of Pharmacy and BioTechnology, University of Bologna, Via S. Donato 19/2, 40127 Bologna, Italy; luisastella.dolci2@unibo.it; 4 Health Sciences and Technologies—Interdepartmental Center for Industrial Research (HST-ICIR), Alma Mater Studiorum-Università di Bologna, Ozzano dell’Emilia, 40064 Bologna, Italy; 5 Interdepartmental Center for Industrial Research on Advanced Applications in Mechanical Engineering and Materials Technology, CIRI-MAM, University of Bologna, Viale Risorgimento, 2, 40136 Bologna, Italy; 6 Osteoncology, Bone and Soft Tissue Sarcomas and Innovative Therapies Unit, IRCCS Istituto Ortopedico Rizzoli, 40136 Bologna, Italy; toni.ibrahim@ior.it

**Keywords:** composite scaffold, electrospinning, hydrogel, PEOT/PBT, gelatin, dual-drug delivery systems, sarcoma, regenerative medicine, chemotherapy

## Abstract

In the clinical management of solid tumors, the possibility to successfully couple the regeneration of injured tissues with the elimination of residual tumor cells left after surgery could open doors to new therapeutic strategies. In this work, we present a composite hydrogel–electrospun nanofiber scaffold, showing a modular architecture for the delivery of two pharmaceutics with distinct release profiles, that is potentially suitable for local therapy and post-surgical treatment of solid soft tumors. The composite was obtained by coupling gelatin hydrogels to poly(ethylene oxide)/poly(butylene terephthalate) block copolymer nanofibers. Results of the scaffolds’ characterization, together with the analysis of gelatin and drug release kinetics, displayed the possibility to modulate the device architecture to control the release kinetics of the drugs, also providing evidence of their activity. In vitro analyses were also performed using a human epithelioid sarcoma cell line. Furthermore, publicly available expression datasets were interrogated. Confocal imaging showcased the nontoxicity of these devices in vitro. ELISA assays confirmed a modulation of IL-10 inflammation-related cytokine supporting the role of this device in tissue repair. In silico analysis confirmed the role of IL-10 in solid tumors including 262 patients affected by sarcoma as a negative prognostic marker for overall survival. In conclusion, the developed modular composite device may provide a key-enabling technology for the treatment of soft tissue sarcoma.

## 1. Introduction

Local drug delivery systems are promising tools in modern medicine because they can assure the release of drugs with the kinetics required by specific applications and a reduction in undesired side effects that are typical of systemic therapies.

In order to be employable as drug carriers for local therapies and tissue regeneration, polymeric scaffolds need to meet specific requirements. In particular, they should be able to incorporate drugs to favor release with predictable kinetics [1,2], to guarantee the retainment of the therapeutics at the site of interest [1], to be biocompatible [3], and to mimic the morphological, chemical, and mechanical properties of the tissues in which they are implanted [4,5,6]. 

Over the years, a wide variety of polymeric scaffolds has been investigated as implantable single drug delivery systems. Some of the adopted approaches exploited drug diffusion through polymeric materials [7]; other strategies focused on the realization of drug-eluting scaffolds, obtained by using properly selected biodegradable polymers, showing a degradation rate able to ensure a sustained release of the drug for a specific period [8,9]; finally, the development of scaffolds able to release the drug when triggered by an external stimulus was also investigated [10]. Among these systems, BCNU wafers (carmustine wafers, Gliadel) have been demonstrated to represent an effective way for the delivery of chemotherapy directly to intracerebral high-grade tumors, with no systemic toxicity [11]. Clinical trials have documented the suitability of Gliadel in a selected group of patients with malignant gliomas. Furthermore, the possibility to improve BCNU activity by administrating it together with agents able to overcome BCNU resistance or with other therapeutics [12] paves the way to the use of Gliadel for the treatment of other intracranial neoplasms and metastatic disease [11]. 

In the last ten years, great efforts have also been devoted to the design of polymeric scaffolds for the release of multiple active compounds from the same device with distinct kinetics [13,14,15] and some of them have also been tested in vivo [16,17,18].

Among the still poorly investigated biomedical applications, postsurgical treatment of solid tumors, including soft tissue sarcoma, is one of the medical areas that could benefit from the employment of implantable drug delivery devices. 

Soft tissue sarcomas are a rare group of solid lesions of mesenchymal origin with uncertain etiology and difficult classification [19]; they account for around 1% of all solid neoplasms [20] and include more than 70 different histological subtypes. They can arise from several districts of the body and their clinical behavior could vary from indolent to very aggressive. They often occur as a soft, painless mass, which becomes painful when pressed on nerves or muscles; thus, their diagnosis is often achieved when they are a large lesion in size [21]. For the above reasons, although surgery represents the mainstay of treatment for localized disease, local or distant postsurgery relapse could be observed depending on several factors including tumor grade, histology, and resection margins free or not from the disease [22]. In this regard, margins status represents a prognostic factor for the overall survival of patients affected by soft tissue sarcoma and the achievement of a radical resection represents a critical point in the management of these diseases. Moreover, due to the huge masses intraoperatively harvested, surgery can produce extended injury in the normal tissue, which needs to be restored. Considering these urgent clinical needs, a primary directive of regenerative medicine is to develop drug-delivery platforms that can achieve the radicalization of solid lesion surgery and promote tissue regeneration.

For the development of implantable scaffolds, the correlation between structure and properties of the employed polymers plays an extremely important role. The system poly(ethylene oxide)/poly(butylene terephthalate) (PEOT/PBT), availing the tuning of its chemical composition, is ideal for the design of implantable scaffolds with desired properties. PEOT/PBT is a class of multiblock thermoplastic copolymers, composed of soft PEOT and hard PBT units. PEOT derives from PEO repetitive units connected to terephthalic units through ester bonds. By acting on copolymer molecular weight, soft to hard segments ratio, and molecular weight of PEO starting unit, the synthesis of polymers showing tunable physical and chemical properties can be achieved [23]. Regarding thermo-mechanical properties, all PEOT/PBT copolymers are semicrystalline at room temperature and show a higher melting temperature and heat of fusion with the increase in PBT content. Furthermore, the elastic modulus significantly decreases with an increase in the content of soft segments. The increase in PEO content also enhances the hydrophilicity of the resulting copolymer and, in tune with this, hydrolytic degradation is faster for copolymers obtained from higher PEO unit molecular weight as a consequence of higher water uptake. Moreover, hydrolysis occurs preferentially in the amorphous phase; therefore, the decrease in PBT segment contents renders the resulting copolymer more hydrolyzable [23]. Although PEOT/PBT copolymers have been widely employed since 1994 [24,25,26,27,28,29,30,31,32,33,34] for applications in the field of tissue regeneration, their potential for the design of drug-releasing scaffolds is still poorly investigated. Indeed, the few studies reported in this context mainly focus on the development of PEOT/PBT scaffolds for the single release of dies [35] and small molecules, such as proteins [36], highlighting, in this case, a release kinetic dependent on the PEO content [36].

Electrospinning represents a widely investigated technique for the development of drug-containing scaffolds for biomedical applications, such as wound dressing, tissue remolding, and the prevention of anaerobic bacteria colonization [37,38]. However, the integration of various antitumor drugs showing different hydrophobic/hydrophilic properties into one electrospun platform, enabling a dual-drug release, is still challenging [37]. In this respect, strategies based on the fabrication of hybrid composite scaffolds, obtained from the combination of electrospun fibers with hydrogels, are raising great interest in the biomedical field [4,39,40,41,42,43,44,45]; moreover, recently, composite nanofiber–hydrogel scaffolds have also been tested for the delivery of proteins and nucleic acid therapeutics in the in vivo treatment of spinal cord injuries [46].

**Figure 1:**
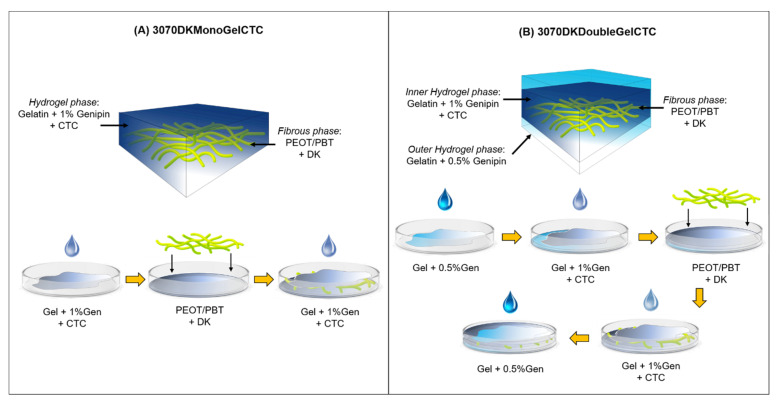
Schematic illustration of the final structure and of the procedure for the fabrication of 3070DKMonoGelCTC (**A**) and 3070DKDoubleGelCTC (**B**).

In this work, we propose the development of a modular composite hydrogel–electrospun scaffold for the delivery of two pharmaceutics with distinct release profiles over time. Nanofibrous mats obtained from PEOT/PBT copolymers have been coupled with gelatin hydrogels to obtain biocompatible hybrid scaffolds with a modulable architecture properly designed to enable the dual drug release. As model drugs, Diclofenac (DK) and Chlorotetracyline hydrochloride (CTC) were used. DK, in the form of potassium salt, was loaded into the electrospun non-woven fabric as an anti-inflammatory drug, while CTC, an antibiotic belonging to the family of tetracyclines, was incorporated within the gelatin hydrogel as a model drug for the anticancer agent Epirubicin. Two hydrogel/fiber hybrid composite scaffolds containing DK and CTC, i.e., mono-layer and double-layer composites (Figure 1) were proposed, differing in terms of gelatin crosslinking degree and number of gelatin layers wrapping the fibrous mat. The complete characterization of the composite systems and the effect of their architecture on the modulation of dual drug release are presented and discussed.

## 2. Results and Discussion

In this work, we developed a modulable platform for dual drug delivery in local therapy. Potentially suitable for several medical applications, the proposed model scaffold could represent an alternative to chemotherapy for the post-surgical treatment of solid soft tumors, such as sarcoma, which could overcome the undesired consequences typical of the common systemic therapies. 

To meet the drug delivery requirements of this application, the scaffold should enable a quick release of an anti-cancer drug to induce the elimination of residual cancerous cells in the surgical site and the sustained delivery of an anti-inflammatory drug to reduce inflammation, also promoting tissue regeneration in the implantation site. In light of these aspects, the model hybrid scaffolds were designed by introducing the anti-inflammatory drug DK inside the nanofibers, while CTC, a model molecule of Epirubicin, was loaded in hydrogel layers. Two distinct release kinetics of these drugs were tuned by acting on the scaffold’s layered structure and on the crosslinking extent of gelatin hydrogel. In particular, the DK release was investigated as a function of both the PEOT/PBT composition and the modulation of the composite’s architecture, while CTC release was controlled by acting on the crosslinking extent of the hydrogel. 

### 2.1. Characterization of PEOT/PBT Electrospun Mats

**Figure 2:**
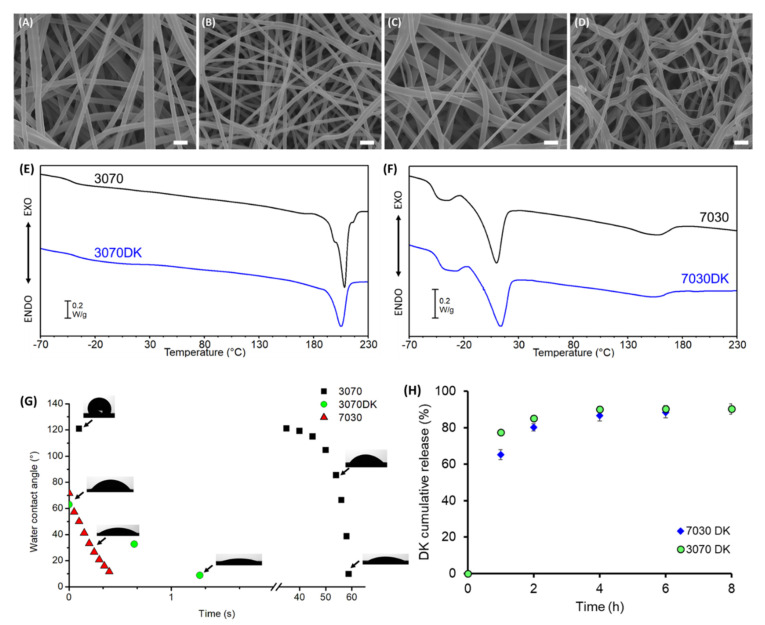
Characterization of the PEOT/PBT electrospun fibers. SEM images of the following: (**A**) 3070, (**B**) 3070DK, (**C**) 7030 and (**D**) 7030DK fibers; scale bar = 10µm. (**E**) DSC curves (second heating scans) of 3070 (black) and 3070DK (blue) mats. (**F**) DSC curves (second heating scans) of 7030 (black) and 7030DK (blue) mats. (**G**) Water contact angle measurements over time for 3070 (black square), 3070DK (green circle) and 7030 (red triangle) mats. (**H**) DK cumulative release profile over time for 3070DK (green circle) and 7030DK (blue diamond) electrospun fabrics.

Two copolymers of PEOT/PBT that differ in composition, namely PEOT70PBT30 and PEOT30PBT70, were employed for the fabrication of nanofibrous mats for the release of DK. To assess the effect of DK on fiber properties, both plain and DK-loaded mats were prepared. The morphological analysis of plain and DK-loaded electrospun mats revealed the presence of regular, bead-free, defect-less fibers in a random arrangement, as shown in Figure 2A–D. Plain mats show fibers diameters in the submicrometric range, 760 (±150) nm for 3070 and 870 (±260) nm for 7030 (Figure S1A,C). In the presence of DK, thinner fibers (Figure S1B,D) were obtained regardless of the employed copolymer with a mean diameter of 500 (±80) nm for 3070DK and 500 (±90) nm for 7030DK: this result can be ascribed to an increase in the polymeric solution’s conductivity as a consequence of the addition of the drug in salt form [47].

The thermogravimetric analysis (Figure S2 and Table S1) highlighted the lack of differences between the two copolymers, both showing a single weight loss around 390 °C. The presence of DK in the mats anticipates the onset of degradation at about 270 °C, consistent with the degradation path of the pure drug. 

As documented by previous studies, the two copolymers are characterized by significantly different thermal transitions, due to their different chemical composition [23]. DSC first heating scans, reported in Figure S3 and Table S2, are all affected by the presence of absorbed water, with a different extent depending on hydrophilic PEOT content and on the presence of DK in line with TGA data.

The second calorimetric heating scan (Figure 2E,F), performed after quenching, clearly reported the presence of a single glass transition temperature (T_g_) well below room temperature (Table S3) ascribable to PEOT soft segments for all the considered mats [23]. Concerning 3070 and 3070DK electrospun mats (Figure 2E), only a melting endothermal peak around 210 °C was detected, highlighting the capability of PBT to crystallize during quenching [23]. Conversely, no crystallization and/or melting transitions ascribable to PEOT segments were detected, probably due to their low content. For 7030 and 7030DK mats (Figure 2F), T_g_ was followed by cold crystallization and melting occurring at −25 °C and 10 °C, respectively, both assigned to PEOT [23]. Additionally, a second melting was detected at 150 °C, demonstrating the capability of the PBT units to generate a small amount of crystalline phase (ΔH_m_= 8 J g^−1^) even when present in low amounts (30%). Finally, the addition of DK in the fibers has the main effect of slightly suppressing the crystallization of PBT blocks in both copolymers, testified by the lower values of the corresponding ΔH_m_.

The water contact angle measurements (Figure 2G) highlighted a remarkable different behavior of the two plain mats: the 7030 fibers, richer in hydrophilic PEOT units, absorbed the water drop in less than 1 s, whereas the more hydrophobic 3070 fibers needed 1 min to be wet. The addition of DK accelerated water drop absorption, which occurred practically instantaneously in 7030DK (not reported) and within a few seconds for 3070DK. The high wettability of both DK-containing mats supported the absence of significant differences in terms of drug release kinetic between the two mats, as documented in Figure 2H. 

A fundamental requirement for the obtainment of interpenetrated hybrid hydrogel-nanofiber composites is the preservation of the fibrous morphology when the mat is placed in contact with the hydrogel aqueous solution; therefore, the typology of the mat to be employed for the preparation of composites was selected based on its water stability.

The morphological analysis of 3070DK and 7030DK performed after overnight immersion in distilled water highlighted that 3070DK mats well retained fibrous morphology, while 7030DKs were subjected to swelling with a partial loss of mat porosity, as shown in Figure S4. Therefore, only 3070DK mat and 3070 mat as controls were employed for the preparation of the composites.

### 2.2. Hydrogel/Nanofibers Hybrid Composites

**Figure 3:**
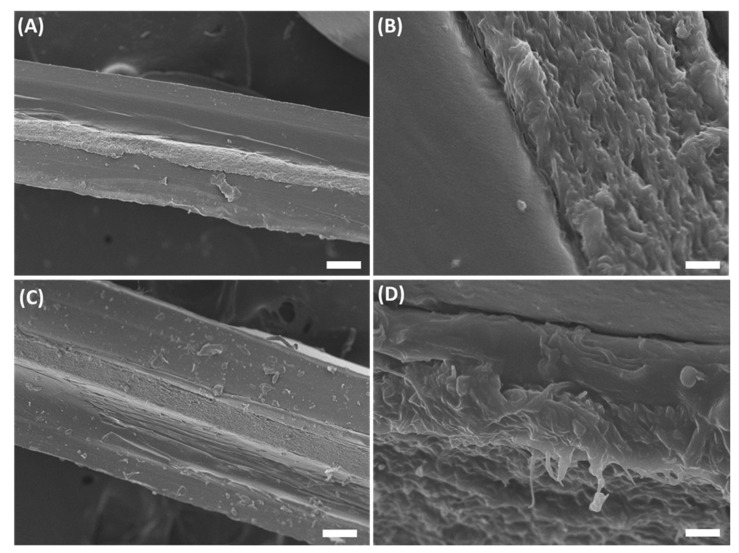
Scanning electron microscopy micrographs of mono-layer (**A**,**B**) and double-layer (**C**,**D**) composites. Scale bar: 100 µm (**A**,**C**), 5 µm (**B**,**D**).

The high hydrophilicity conferred by DK to 3070 mats enabled their good impregnation with the gelatin-based hydrogel, as confirmed by SEM cross-sectional analysis carried out on 3070DKMonoGelCTC and 3070DKDoubleGelCTC hybrid composites (Figure 3), whose fabrication procedure is shown in Figure 1 and described in Materials and Methods. Indeed, as observable in Figure 3B,D, the hydrogel completely fills the pores of the 3070DK mat without affecting its fibrous texture. Furthermore, as shown in Figure 3C, in double-layer composites, the hydrogel phase appeared as a single continuous layer, making the separation between the two distinct gelatin-based hydrogels barely detectable (Figure 3D). Taken together, the results of SEM analysis demonstrated a good interconnection between the different layers of the scaffolds. 

**Figure 4:**
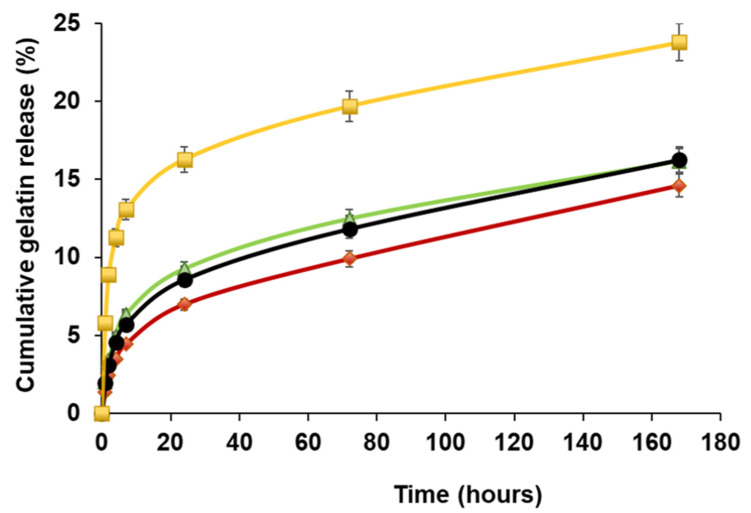
Gelatin cumulative release from 3070DKMonoGelCTC (yellow), 3070DKDoubleGelCTC (green), 3070MonoGel (black) and 3070DoubleGel (red). Each analysis was carried out in triplicate.

All tested composites (3070MonoGel, 3070DKMonoGelCTC, 3070DoubleGel, 3070DKDoubleGelCTC) neither broke nor delaminated after immersion in PBS at 37 °C for 1 week. Scaffold stability was evaluated by gelatin release as a function of storage time in PBS and, as reported in Figure 4, mono-layer drug-loaded composites showed a faster gelatin release with respect to both plain and 3070DKDoubleGelCTC devices. Moreover, statistical analysis confirmed a significantly higher gelatin release from 3070DKMonoGelCTC with respect to 3070DKDoubleGelCTC (**** *p* < 0.001 at 24, 72, and 168 h). This result might be ascribed to the interference of CTC in the gelatin crosslinking. Indeed, by lowering the pH of the solution [48], CTC hindered the crosslinking process, thus enhancing gelatin solubility. In tune with this hypothesis, the presence in the double-layer composite of an additional hydrogel layer not containing CTC increased the stability of the tested samples and, therefore, slowed down the gelatin release with respect to the mono-layer. Accordingly, the amount of gelatin released from 3070DKDoubleGelCTC is higher with respect to that released from the plain double-layer composite (**** *p* < 0.0001 at 24, 72, and 168 h) in which the extent of crosslinking is not affected by drug addition. The composite 3070DoubleGel turned out to be the most stable over time, also compared with 3070MonoGel (*** *p* < 0.001 at 24 h and **** *p* < 0.0001 at 72 and 168 h), thanks to the presence of an additional gelatin layer.

### 2.3. Drugs Release

To investigate the drugs’ release profiles as a function of the device’s composition, we first separately studied DK and CTC drug profiles from mono-layer and double-layer composites. The release of DK from electrospun fibers was also reported as a comparison. 

**Figure 5:**
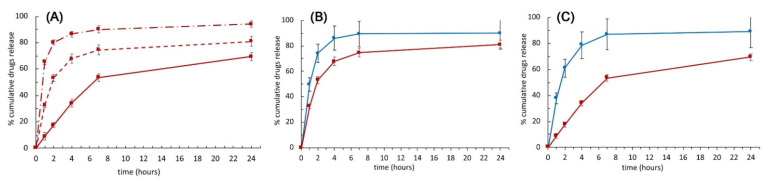
(**A**) Drugs cumulative releases. DK cumulative release profile over time from fibers (dash-dot line), mono-layer (dotted line), and double-layer composites (continuous line). (**B**) Comparison between CTC (blue) and DK (red) cumulative release profiles over time from mono-layer (**B**) and double-layer composite devices (**C**), respectively.

As shown in Figure 5A, DK was released from fibers with an evident high initial burst; indeed, around the 80% *w/w* of the drug was released during the first 2 h of incubation and residual DK was completely released within 4 h. Interestingly, a significantly different behavior was observed for mono-layer and double-layer composite scaffolds for which DK releases around 55% and 20%, respectively, were achieved after 2 h of incubation. Furthermore, after the first 24 h, only 85% *w/w* and 65% *w/w* of DK were delivered from the mono-layer and double-layer devices, respectively. These percentages became approximately 90% *w/w* and 78% *w/w* after three days of incubation for the two systems, respectively.

In light of these results, the presence of the hydrogel layer obtained from Gel1 solution significantly contributed to slowing down DK’s release rate in double-layer composite, acting as an effective diffusion barrier. This achievement suggested the possibility to tune the thickness of the hydrogel layer to reach the desired release kinetic for the drug contained in the electrospun fibers. 

As observable by comparing Figure 5B,C, CTC release from the two devices showed the same dramatically fast initial burst release of the drug within the first hour. In addition, regardless of the genipin concentration used for gelatin crosslinking, almost all the drugs are released from the device in 4 h. A slight low initial burst release is observed in the double layer probably due to the diffusion of the drug inside the Gel1 layer.

The profiles of DK and CTC when contextually released from 3070DKMonoGelCTC and 3070DKDoubleGelCTC are reported in Figure 5B,C, respectively. For mono-layer composites, statistical analysis highlighted a slightly slower release of DK with respect to CTC, with significant differences at 1 h (* *p* < 0.05), 2 h (** *p* < 0.01), 4 h (** *p* < 0.01), and 7 h (* *p* < 0.05); conversely, no statistically significant differences were observed at 24 h. 

Concerning the double-layer platform, it turned out to simultaneously ensure a fast release of the CTC loaded in the outer hydrogel layers and a sustained release of the DK contained in the electrospun nanofibers. Indeed, for the time points from 1 h to 7 h, significant differences between the release kinetics of the two drugs were obtained with **** *p* < 0.0001; the significant difference was maintained also at 24 h with a ** *p*-Value< 0.01.

### 2.4. In Vitro Viability Assays

**Figure 6:**
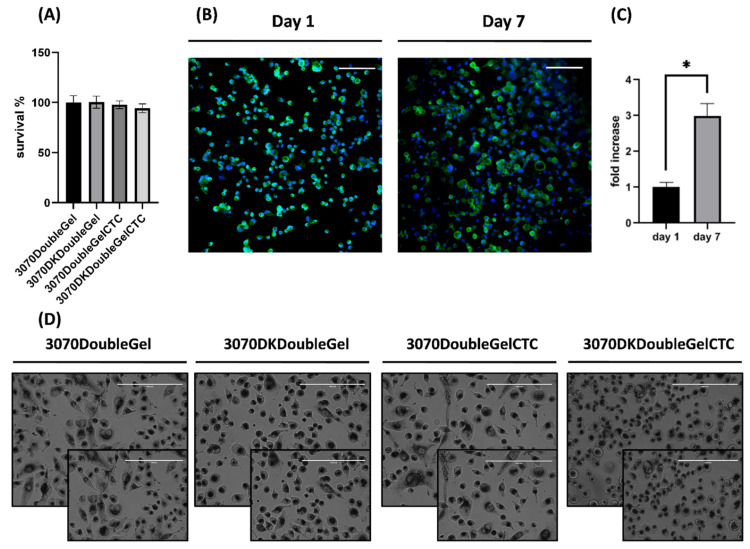
(**A**) Viability assay of VA-ES-BJ human epithelioid sarcoma cell line in a transwell culture system (indirect co-culture) with 3070DoubleGel, 3070DKDoubleGel, 3070DoubleGelCTC, and 3070DKDoubleGelCTC. (**B**) Representative immunofluorescence confocal images of VA-ES-BJ human epithelioid sarcoma cell line cultured in an empty patch at days 1 and 7. Actin filaments were stained with phalloidin (green) and nuclei were counterstained with dapi (blue). Magnification at 20×, scale bar = 200 µm. (**C**) Fold change in cell proliferation (cell number) day 7 versus day 1 (* *p* ˂ 0.05). (**D**) Morphological characterization of VA-ES-BJ human epithelioid sarcoma cell line after exposure to patches in the indirect co-culture. Architectural features of VA-ES-BJ with typical epithelial-appearing (ovoid or polygonal) cells mixed with fusiform cells with many intracytoplasmic vacuoles were maintained with 3070DoubleGel, 3070DKDoubleGel, and 3070DoubleGelCTC while morphological changes such as rounding up were observed with a combination treatment (3070DKDoubleGelCTC). Magnification 20×, scale bar = 200 µm.

In vitro viability assays were carried out on the following double-layer devices: composite scaffolds not containing drugs (3070DoubleGel) as the control, composite scaffolds containing only DK (3070DKDoubleGel), composite scaffolds containing only CTC (3070DoubleGelCTC), and composite scaffolds containing both DK and CTC (3070DKDoubleGelCTC). To evaluate the impact of all considered materials on tumor cell viability, an indirect co-culture was carried out. The results showed that no significant differences between empty-loaded and drug-loaded scaffolds were observed in tumor cells viability (Figure 6A). The spindle shape morphology of VA-ES-BJ human epithelioid sarcoma cell line was maintained when the cells were exposed to DK and CTC in monoregimen (Figure 6D). A non-significant difference in cell viability was observed between single loaded scaffolds and 3070DKDoubleGelCTC, which achieved a slight decrease compared to the control group; this result was sustained by the appearance of round cells in the culture (Figure 6D). In this regard, a possible explanation could be related to the combination of these drugs and their metabolites, which could affect the viability and morphology of VA-ES-BJ sarcoma cells. Since the tested drugs (DK, CTC, and DK + CTC) do not exert cytotoxic effects, the observed results supported their molecular mechanism. On the other hand, although these drugs do not impair cell survival, a slight decrease in cell viability was achieved compared to the control condition, providing, as explained above, indirect evidence of the ability of the device in drug delivery. Furthermore, the not-observed decrease in cell viability with the control device suggests the biosafety of PEOT/PBT nanofibers and gelatin hydrogel.

### 2.5. Non-Toxicity Profile

To further strengthen the biocompatibility of the devices tested in in vitro survival assays and to corroborate the obtained results, a confocal analysis at two-time points was performed. The results clearly showed that the devices were able to sustain the viability of cells (Figure 6B), as observed by the significant fold increase between 24 h and 7 days post-cell seeding (Figure 6C). In this regard, the cell number was triplicated in six days of culture. Furthermore, no morphological changes were detected in nuclei and actin filaments of the cell culture, corroborating the non-toxicity profile of the device. A study limitation is represented by the missing experiments on health cells, which should be carried out in future analysis.

### 2.6. Regenerative Medicine Approach

**Figure 7:**
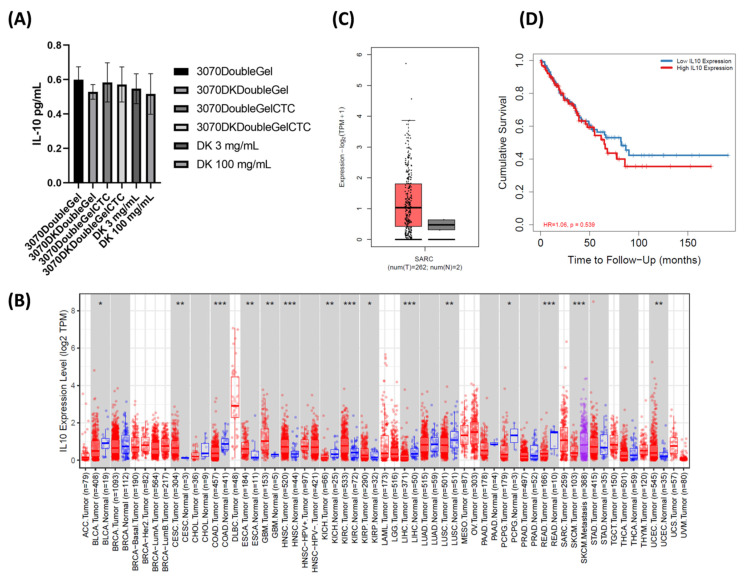
(**A**) IL-10 protein detection by using an ELISA assay in the supernatant of VA-ES-BJ human epithelioid sarcoma cell line in a transwell culture system (indirect co-culture) with3070DoubleGel, 3070DKDoubleGel, 3070DoubleGelCTC, 3070DKDoubleGelCTC, and positive control DK at both the concentration of 3mg mL^−1^ (human plasma peak) and 100 mg mL^−1^ (expected cumulative release from the device). (**B**) In silico analysis of IL-10 mRNA expression among tumors (red bar) and normal tissues (blue bar, reported when available), *: *p*-value < 0.05; **: *p*-value <0.01; ***: *p*-value <0.001. (**C**) IL-10 mRNA expression among 262 patients affected by sarcoma (red bar) and normal tissues (grey bar). (**D**) Kaplan–Meier curve of overall survival analysis based on the expression status of IL-10 in multiple cancer types.

The ability of the scaffolds in reducing tumor cell inflammation was investigated by using an ELISA assay. In this regard, IL-10, a well-known pro-inflammatory and anti-inflammatory pleiotropic cytokine, is one of the involved targets of anti-inflammatory drugs for DK. The expected result in decreasing the presence of IL-10 was confirmed by a trend of significant modulation of IL-10 detected in the supernatant of indirect co-culture compared to the other conditions (Figure 7A). Moreover, the observed results were comparable to that of the positive control of the tested free DK at a concentration of 3 mg mL^−1^, which represents human plasm peak concentration [49]. Furthermore, a higher decrease in IL-10 detection was observed in the positive control at a concentration of 100 mg mL^−1^, which represents the estimated cumulative release. These latter data provide support for the fidelity of the analysis and underline the ability of the device to perform an effect similar to that due to the free drug at the same human plasma peak concentration. Taken together, these results provide evidence of the potential role in regenerative medicine of this device. 

### 2.7. IL-10 Is Associated with Poor Prognosis in Solid Tumors

To confirm the robustness of the obtained data, publicly available expression datasets on IL-10 were investigated. Firstly, the expression of this marker was analyzed among several solid tumors. The results confirmed a higher expression of this marker among tumors compared to normal tissue (Figure 7B). The investigation of IL-10 expression in 262 patients affected by sarcoma confirmed the higher expression of IL-10 compared to normal tissue (Figure 7C). Finally, the expression of this marker was correlated to the patient’s clinical outcome. In this regard, the results showed IL-10 as a negative prognostic factor for overall survival (Figure 7D), confirming its role in solid tumors. These results further corroborate the role of IL-10 in solid tumors, including sarcoma, and support the rationale of targeting this marker as a valuable therapeutic option in a clinical setting.

## 3. Conclusions

The efficacy of composite scaffolds made of PEOT/PBT nanofibers and gelatin hydrogel for the simultaneous release of two drugs with two distinct kinetics was confirmed. We proved that the dual loading of drugs in this modular composite device is feasible and could provide therapeutic advantages. In vitro analysis showed the ability of drug release exerted by the device, providing the rationale for using chemotherapeutics. Moreover, the non-toxicity profile of the scaffolds was confirmed by a viability assay and morphological and confocal imaging analyses. Furthermore, we demonstrated the ability of the device in modulating the secretion of inflammation-associated cytokine IL-10. Finally, in silico analysis confirmed the role of IL-10 as a negative prognostic marker for solid tumors in overall survival. This smart device could represent a new groundbreaking strategy for the treatment of soft tissue sarcoma.

## 4. Materials and Methods

### 4.1. Materials

Two distinct poly(ethylene oxide)terephthalate/poly(butylene terephthalate) (PEOT/PBT) block copolymers, 1000PEOT30PBT70 and 1000PEOT70PBT30, were purchased from PolyVation (Groningen, The Netherlands). The copolymers are labeled as *a*PEOT*b*PBT*c*, in which *a* is the PEO molecular weight, *b* is the weight percentage of PEO-therephtalate (PEOT), and *c* is the weight percentage of PBT. Type A Gelatin 300 Bloom (Sigma Aldrich, Milan, Italy) from porcine skin was used. Genipin (min. 98%) was supplied by Wako Chemicals Europe GmbH (Neuss, Deutschland). Diclofenac Potassium (DK) was provided by Farmalabor (Assago, Milan, ITALY). Chlorotetracycline Hydrochloride (CTC), 1,1,1,3,3,3-Hexafluoro-2-propanol (HFP ≥ 99%), bicinchonininc acid solution, and copper (II) sulfate pentahydrate were purchased from Sigma-Aldrich (Milan, ITALY) and used without further purification.

### 4.2. Fabrication of Electrospun Mats

PEOT/PBT copolymer (either 1000PEOT30PBT70 or 1000PEOT70PBT30) was dissolved in HFP at a concentration of 20% *w/v* and stirred at room temperature. DK, at a concentration of 5% *w/w* with respect to the weight of PEOT/PBT copolymer, was previously solubilized in an aliquot of HFP and added dropwise to the polymer solution. The resulting solution was kept under stirring for 1 h before the electrospinning. The electrospinning process was carried out by using a homemade electrospinning apparatus composed of a high voltage power supply (Spellman, SL 50 P 10/CE/230), a syringe pump (KD Scientific 200 series), a glass syringe containing the polymer solution, a stainless-steel blunt-ended needle (inner diameter = 0.5 mm) connected to the power supply, and a grounded cylindrical aluminum collector (rotation angular speed = 60 rpm). The polymer solution was dispensed through a PTFE tube to the needle, which was placed at a distance of 20 cm from the collector. The process was performed at 25 °C and a relative humidity of 50% with a solution flow rate of 1.2 mL h^−1^ and an applied voltage of 19 kV DC. Mats with a thickness of around 60 μm were obtained and stored overnight in a desiccator to remove residual solvents. The resulting electrospun mats obtained from 1000PEOT70PBT30 and 1000PEOT30PBT70 were labeled 7030DK and 3070DK, respectively. Electrospun mats not containing DK were also produced for comparison and labeled 7030 and 3070, respectively. 

### 4.3. Fabrication of the Hydrogels

The following gelatin-based solutions were considered for the preparation of the hydrogels: “gelatin solutions”, containing gelatin and genipin as crosslinking agent; “gelatin-CTC solutions”, containing gelatin, genipin and CTC. “Gelatin solutions” were obtained according to the following steps: (i) gelatin (10% *w/v*) was dissolved in distilled water at 45 °C under stirring; (ii) genipin (either 1% or 0.5% *w/w* with respect to gelatin) was dissolved in phosphate-buffer solution (PBS, 0.1 M, pH 7.4) at 42 °C under stirring for 30 min; (iii) the two solutions were mixed with a volumetric ratio H2O:PBS = 9:1 and kept under stirring at 45 °C for 3–5 min. “Gelatin-CTC solutions” were produced by the following: (i) adding previously grinded CTC powder (2% *w/w* with respect to gelatin) to the gelatin solution under stirring at 45 °C for 3 min and keeping the mixture protected from light [50,51]; (ii) adding to the obtained solution the genipin solution prepared as previously described; (iii) keeping the mixture under stirring at 45 °C for 3–5 min. “Gelatin-CTC solutions” containing genipin at a concentration of either 1% or 0.5% *w/w* were labeled Gel1CTC and Gel0.5CTC, respectively. Similarly, “gelatin solutions” were labeled Gel1 and Gel0.5.

### 4.4. Preparation of Hydrogel/Fibers Hybrid Composite Scaffolds

For the preparation of hydrogel/fibers hybrid composite scaffolds, 3070DK and 3070 electrospun mats were employed. Two types of scaffolds, defined mono-layer and double-layer composites were obtained following the procedure depicted in Figure 1. For the preparation of monolayer composites, 4 mL of Gel1CTC was poured in a Petri dish (inner diameter = 5 cm) and kept at room temperature for 10 min; then the electrospun sample, rectangular-shaped (0.5 × 4 cm^2^), was placed onto the gelatin layer and covered with additional 4 mL of Gel1CTC (Figure 1A). The scaffolds were labeled 3070DKMonoGelCTC. Double-layer composites, labeled 3070DKDoubleGelCTC, were produced as follows (Figure 1B): (i) 2 mL of Gel0.5CTC was poured in a Petri dish and allowed to gel; (ii) 4 mL of Gel1 solution was laid down over this layer; (iii) after gelification, the electrospun mat was placed onto the hydrogel; (iv) the mat was covered by a double layer made of 4 mL of Gel1 and 2 mL of Gel0.5CTC following procedure (ii) and (i). The resulting mono-layer and double-layer composites were kept at 4 °C for 24 h, and the scaffolds were obtained from the solvent casting method after solvent evaporation for 24 h at RT under a laminar flow hood. Scaffolds not containing drugs were produced for comparison and labeled 3070MonoGel and 3070DoubleGel, respectively. Square-shaped samples (1 × 1 cm^2^) for the evaluation of gelatin and drug release were also prepared. 

### 4.5. Characterization Techniques

Thermogravimetric analysis (TGA) of the electrospun mats was carried out using a TA Instrument TGA Q500 analyzer from RT to 900 °C, with a high-resolution dynamic mode at 50 °C min^−1^ and a resolution index of 5 in N_2_ atmosphere. Differential Scanning Calorimetry (DSC) measurements of the electrospun mats were carried out using a TA Instruments Q100 DSC apparatus in N_2_ atmosphere from −90 °C to 250 °C with a heating scan rate of 20 °C min^−1^; T_g_ was taken at half-height of the glass transition heat capacity step in the second heating scan. Water contact angle (WCA) measurements were performed using the Theta Lite instrument (Biolin Scientific, Alessandria, Italy) equipped with One Attension software. Distilled water was used for the measurements. Ten measurements were performed for each sample, and the water drop profiles were collected in a time range 0–120 s. The morphology of the electrospun fibers and hydrogel/fibers hybrid composite scaffolds was investigated by using a Philips 515 Scanning Electron Microscope (SEM); samples were sputter-coated with gold before examination and the distribution of fiber diameters was determined with measurements of about 300 fibers by employing image analysis software (EDAX Genesis).

### 4.6. Determination of the Gelatin Release

For the gelatin release evaluation, square-shaped (1 × 1 cm^2^) mono-layer and double-layer composite samples were immersed in 10 mL of PBS at 37 °C. After each time point (from 1 h to 7 days), PBS was removed and replaced with a fresh solution. Gelatin concentration in the release buffer was determined by a colorimetric method using the bicinchoninic acid protein assay, following the previously reported procedure [52,53]. Each analysis was carried out in triplicate. The cumulative gelatin release (%) was calculated with respect to the starting weight of each sample. 

### 4.7. Drug Release

Square-shaped (1 × 1 cm^2^) mono-layer and double-layer composites were immersed in 10 mL of PBS at 37 °C. For each time point (from 1 h to 7 days), PBS was withdrawn and replaced with a fresh solution. The quantification of DK and CTC in the solution was carried out using a HPLC-UV/Vis apparatus (equipped with Jasco PU2089 Plus pump and Jasco MD-2010 Plus detector). An autosampler (SIL-20A, Shimadzu, Japan) was used to inject samples (20 μL) onto a C18 column (15 cm × 4.6 mm × 5 μm, Phenomenex). The flow rate was 1 mL min^−1^ and the detection wavelength was set for DK and CTC at 220 nm and 260 nm, respectively. For DK quantification, a mobile phase of acetonitrile: ammonium phosphate buffer (20 mM, pH 2.5) = 70:30 (*v/v*) was used. The running time and drug retention time were set at 10 min and 4.2 min, respectively. CTC quantification was performed by using a mobile phase of acetonitrile:ammonium phosphate buffer (20 mM, pH 2.5) = 80:20 (*v/v*). In this case, the running time and retention time were set at 15 min and 6.4 min, respectively. Both methods required the preparation of calibration curves by using standard solutions ranging from 0.1 to 20 μg mL^−1^ in PBS (DK: y = 29486x + 4785.5, R2 = 1; CTC: y = 19140x + 1982.4, R2 = 0.999).

### 4.8. Cell Seeding and Culture

The experiments were performed on a VA-ES-BJ human epithelioid sarcoma cell line obtained from the America Type Culture Collection (Rockville, MD, USA). The cells were cultured as a monolayer in 75 cm^2^ flasks at 37 °C in a 5% CO_2_ atmosphere in DMEM medium supplemented with 10% fetal bovine serum, 1% penicillin-streptomycin, and 1% glutamine (PAA, Piscataway, NJ, USA) referred to as complete DMEM.

### 4.9. Cell Viability Assay

For proliferation profiling, 5 × 10^5^ VA-ES-BJ were plated in 6-well plates. The cells were allowed to adhere and, after 24 h, 24 mm diameter transwell inserts with 0.4 µm pores (Corning Ltd., Flintshire, UK) containing hydrogel/fibers hybrid composite scaffolds (3070DoubleGel, 3070DKDoubleGel, 3070DoubleGelCTC, 3070DKDoubleGelCTC) were placed over VA-ES-BJ cultures and the indirect co-culture was started as already shown [54]. Cells were cultured in 4 mL complete DMEM for 72 h. Transwell inserts with 0.4 µm pores allowed the release of drugs from hydrogel/fibers hybrid composite scaffolds. After exposing tumor cells to the tested conditions, cell viability percentage was assessed by using an MTT reduction assay (Sigma Aldrich), as previously reported [55,56,57]. Experiments were performed in triplicate.

### 4.10. Confocal Analysis

Confocal analysis was performed on hydrogel/fibers hybrid composite scaffolds. Briefly 1 × 10^5^ cells were seeded 3070DoubleGel, as previously described [58]. The cells were washed 3 times with 1% PBS, fixed with 4% PFA for 20 min at room temperature, and stained with DAPI (1:1000, Life Technologies, Carlsbad, CA, USA) and Phalloidin (1:40 Alexa Fluor 488 phalloidin, Life Technologies, Carlsbad, CA, USA). The scaffolds were analyzed after 24 h and 7 days. Images were acquired with an A1 laser confocal microscope (Nikon Corporation, Tokyo, Japan) and analyzed with NIS Elements software (Nikon Corporation, Tokyo, Japan) [59].

### 4.11. ELISA Analysis

The human IL-10 ELISA Kit was used following the manufacturer’s instructions (Sigma Aldrich, Saint Louis, MO, USA). Briefly, cell culture supernatant from the indirect co-culture experiments was harvested, and the levels of IL-10 expression were assayed by using an enzyme-linked immunosorbent assay [60]. All samples were tested in duplicate for the marker.

### 4.12. In Silico Analysis

Gene expression profiling interactive analysis 2 (http://gepia2.cancer-pku.cn/#general, accessed on 18 May 2021) and Tumor IMmune Estimation Resource (TIMER, https://cistrome.shinyapps.io/timer/ accessed on 18 May 2021) were used for the differential expression analysis of IL-10 in tumor and normal tissue from various cancers as previously reported [61].

### 4.13. Statistical Analysis

Statistical analysis was performed with Graph Pad Prism 9 (GraphPad Software Inc., San Diego, CA 92108). A two-way analysis of variance (two-way ANOVA), followed by Bonferroni’s multiple comparison test, was employed to verify the differences between the devices in terms of gelatin and drugs release at the experimentally investigated time points. Differences were considered statistically significant for *p*-values < 0.05. For biological analysis, each experiment was performed in three independent replicates. Data are presented as mean ± standard deviation (SD), or mean ± standard error (SE), as reported. A two-tailed Student’s t-test was used to assess differences between groups, and the results are accepted as significant at *p* < 0.05.

## Data Availability

The data used to support the findings of this study are available from the corresponding author upon reasonable request.

## Supplementary Materials

The following supporting information can be downloaded at: https://www.mdpi.com/article/10.3390/ijms23063239/s1.

## References

[1] Chew S.A., Danti S. (2017). Biomaterial-Based Implantable Devices for Cancer Therapy. Adv. Healthc. Mater..

[2] Maxwell D.J., Hicks B.C., Parsons S., Sakiyama-Elbert S.E. (2005). Development of rationally designed affinity-based drug delivery systems. Acta Biomater..

[3] Williams D.F. (2008). On the mechanisms of biocompatibility. Biomaterials.

[4] Gualandi C., Bloise N., Mauro N., Ferruti P., Manfredi A., Sampaolesi M., Liguori A., Laurita R., Gherardi M., Colombo V. (2016). Poly-l-Lactic Acid Nanofi ber–Polyamidoamine Hydrogel Composites: Preparation, Properties, and Preliminary Evaluation as Scaffolds for Human Pluripotent Stem Cell Culturing. Macromol. Biosci..

[5] Wang X., Ding B., Li B. (2013). Biomimetic electrospun nanofibrous structures for tissue engineering. Mater. Today.

[6] Freedman B.R., Mooney D.J. (2019). Biomaterials to Mimic and Heal Connective Tissues. Adv. Mater..

[7] Stewart S.A., Domínguez-Robles J., Donnelly R.F., Larrañeta E. (2018). Implantable Polymeric Drug Delivery Devices: Classification, Manufacture, Materials, and Clinical Applications. Polymers.

[8] Preethi G.U., Sreekutty J., Unnikrishnan B.S., Archana M.G., Syama H.P., Deepa M., Shiji R., Anusree K.S., Sreelekha T.T. (2020). Doxorubicin eluting microporous polysaccharide scaffolds: An implantable device to expunge tumour. Mater. Sci. Eng. C.

[9] Ranganath S.H., Wang C.-H. (2008). Biodegradable microfiber implants delivering paclitaxel for post-surgical chemotherapy against malignant glioma. Biomaterials.

[10] Cen D., Wan Z., Fu Y., Xu H.P.J., Wang Y., Wu Y., Li X., Cai X. (2020). Implantable fibrous ‘patch’ enabling preclinical chemo-photothermal tumor therapy. Colloids Surf. B Biointerfaces.

[11] Bota A.D., Desjardins A., Quinn J.A., Affronti M.L., Friedman H.S. (2007). Interstitial chemotherapy with biodegradable BCNU (Gliadel®) wafers in the treatment of malignant gliomas. Ther. Clin. Risk Manag..

[12] Ashby L.S., Smith K.A., Stea B. (2016). Gliadel wafer implantation combined with standard radiotherapy and concurrent followed by adjuvant temozolomide for treatment of newly diagnosed high-grade glioma: A systematic literature review. World J. Surg. Oncol..

[13] Xu J., Jiao Y., Shao X., Zhou C. (2011). Controlled dual release of hydrophobic and hydrophilic drugs from electrospun poly(l-lactic acid) fiber mats loaded with chitosan microspheres. Mater. Lett..

[14] Sundararaj S.C., Thomas M.V., Peyyala R., Dziubla T.D., Puleo D.A. (2013). Design of a multiple drug delivery system directed at periodontitis. Biomaterials.

[15] Nagiah N., Murdock C.J., Bhattacharjee M., Nair L., Laurencin C.T. (2020). Development of Tripolymeric Triaxial Electrospun Fibrous Matrices for Dual Drug Delivery Applications. Sci. Rep..

[16] Lee S.S., Kim J.H., Jeong J., Kim S.H.L., Koh R.H., Kim I., Bae S., Lee H., Hwang N.S. (2020). Sequential growth factor releasing double cryogel system for enhanced bone regeneration. Biomaterials.

[17] Lee D., Wufuer M., Kim I., Choi T.H., Kim B.J., Jung H.G., Jeon B., Lee G., Jeon O.H., Chang H. (2021). Sequential dual-drug delivery of BMP-2 and alendronate from hydroxyapatite-collagen scaffolds for enhanced bone regeneration. Sci. Rep..

[18] Fu Y., Cen D., Zhang T., Jiang S., Wang Y., Cai X., Li X., Han G. (2020). Implantable fibrous scaffold with hierarchical microstructure for the ‘onsite’ synergistic cancer therapy. Chem. Eng. J..

[19] WHO (2020). WHO Classification of Tumours. Soft Tissue and Bone.

[20] Siegel R.L., Miller K.D., Jemal A. (2019). Cancer statistics, 2019. CA Cancer J Clin..

[21] Casali P.G., Abecassis N., Aro H.T., Bauer S., Biagini R., Bielack S., Bonvalot S., Boukovinas I., Bovee J.V.M.G., Brodowicz T. (2018). ESMO Guidelines Committee and EURACAN. Soft tissue and visceral sarcomas: ESMO-EURACAN Clinical Practice Guidelines for diagnosis, treatment and follow-up. Ann Oncol..

[22] Gronchi A., Colombo C., Raut C.P. (2014). Surgical management of localized soft tissue tumors. Cancer.

[23] Deschamps A., Grijpa D.W., Feijen J. (2001). Poly(ethylene oxide)/poly(butylene terephthalate) segmented block copolymers: The effect of copolymer composition on physical properties and degradation behavior. Polymer.

[24] Gaillard M.L., Van Blitterswijk C.A. (1994). Pre-operative addition of calcium ions or calcium phosphate crystals to PEOT/PBT copolymers (Polyactive TM) stimulates bone mineralization in vitro. J. Mater. Sci. Mater. Med..

[25] Van Haastert R.M., Grote J.J., Van Blitterswljk C.A., Prewett A.B. (1994). Osteoinduction within PEO/PBT copolymer implants in cranial defects using demineralized bone matrix. J. Mater. Sci. Mater. Med..

[26] Radder M., Van Blitterswijk C.A. (1994). Abundant postoperative calcification of an elastomer: Matrix calcium phosphate-polymer composite for bone reconstruction. J. Mater. Sci. Mater. Med..

[27] Radder A.M., Leenders H., van Blitterswijk C.A. (1995). Bone-bonding behaviour of poly(ethylene oxide)-polybutylene terephthalate copolymer coatings and bulk implants: A comparative study. Biomaterials.

[28] Sakkers R.J.B., Dalmeyer R.A.J., de Wijn J.R., van Blitterswijk C.A. (2000). Use of bone-bonding hydrogel copolymers in bone: An in vitro and in vivo study of expanding PEO-PBT copolymers in goat femora. J. Biomed. Mater. Res..

[29] Deschamps A., Claase M.B., Sleijster W.J., de Bruijn J.D., Grijpma D.W., Feijen J. (2002). Design of segmented poly(ether ester) materials and structures for the tissue engineering of bone. J. Control. Release.

[30] Claase M.B., Riekerink M.B.O., de Bruijn J.D., Grijpma D.W., Engbers G.H.M., Feijen J. (2003). Enhanced Bone Marrow Stromal Cell Adhesion and Growth on Segmented Poly(ether ester)s Based on Poly(ethylene oxide) and Poly(butylene terephthalate). Biomacromolecules.

[31] Carrow J.K., Di Luca A., Dolatshahi-Pirouz A., Moroni L., Gaharwar A.K. (2019). 3D-printed bioactive scaffolds from nanosilicates and PEOT/PBT for bone tissue engineering. Regen. Biomater..

[32] Moroni L., Hendriks J.A.A., Schotel R., De Wijn J.R., Van Blitterswijk C.A. (2007). Design of Biphasic Polymeric 3-Dimensional Fiber Deposited Scaffolds for Cartilage Tissue Engineering Applications. Tissue Eng..

[33] Gonçalves de Pinho R., Odila I., Leferink A., van Blitterswijk C., Camarero-Espinosa S., Moroni L. (2019). Hybrid Polyester-Hydrogel Electrospun Scaffolds for Tissue Engineering Applications. Front. Bioeng. Biotechnol..

[34] Jansen E.J.P., Pieper J., Gijbels M.J.J., Guldemond N.A., Riesle J., Van Rhijn L.W., Bulstra S.K., Kuijer R. (2009). PEOT/PBT based scaffolds with low mechanical properties improve cartilage repair tissue formation in osteochondral defects. J. Biomed. Mater. Res. A.

[35] Moroni L., Licht R., de Boer J., de Wijn J.R., van Blitterswijk C.A. (2006). Fiber diameter and texture of electrospun PEOT/PBT scaffolds influence human mesenchymal stem cell proliferation and morphology, and the release of incorporated compounds. Biomaterials.

[36] Bezemer J.M., Grijpma D.W., Dijkstra P.J., van Blitterswijk C.A., Feijen J. (1999). A controlled release system for proteins based on poly(ether ester) block-copolymers: Polymer network characterization. J. Control. Release.

[37] Ding J., Zhang J., Li J., Li D., Xiao C., Xiao H., Yang H., Zhuang X., Chen X. (2019). Electrospun polymer biomaterials. Prog. Polym. Sci..

[38] Feng X., Li J., Zhang X., Liu T., Ding J., Chen X. (2019). Electrospun polymer micro/nanofibers as pharmaceutical repositories for healthcare. J. Control. Release.

[39] Bosworth L.A., Turner L.A., Cartmell S.H. (2013). State of the art composites comprising electrospun fibres coupled with hydrogels: A review. Nanomed. Nanotechnol. Biol. Med..

[40] Xu S., Deng L., Zhang J., Yin L., Dong A. (2016). Composites of electrospun-fibers and hydrogels: A potential solution to current challenges in biological and biomedical field. J. Biomed. Mater. Res. B Appl. Biomat..

[41] Kim J.H., Choi Y.J., Yi H.G., Wang J.H., Cho D.-W., Jeong Y.H. (2017). A cell-laden hybrid fiber/hydrogel composite for ligament regeneration with improved cell delivery and infiltration. Biomed. Mater..

[42] Mohabatpour F., Karkhaneh A., Sharifi A.M. (2016). A hydrogel/fiber composite scaffold for chondrocyte encapsulation in cartilage tissue regeneration. RSC Adv..

[43] Sadat-Shojai M., Khorasani M.-T., Jamshidi A. (2016). A new strategy for fabrication of bone scaffolds using electrospun nano-HAp/PHB fibers and protein hydrogels. Chem. Eng. J..

[44] Khorshidi S., Karkhaneh A. (2018). A review on gradient hydrogel/fiber scaffolds for osteochondral regeneration. J. Tissue Eng. Regen. Med..

[45] Imere A., Ligorio C., O’Brien M., Wong J.K.F., Domingos M., Cartmell S.H. (2021). Engineering a cell-hydrogel-fibre composite to mimic the structure and function of the tendon synovial sheath. Acta Biomater..

[46] Nguyen L.H., Gao M., Lin J., Wu W., Wang J., Chew S.W. (2017). Three-dimensional aligned nanofibers-hydrogel scaffold for controlled non-viral drug/gene delivery to direct axon regeneration in spinal cord injury treatment. Sci. Rep..

[47] Zong X., Kim K., Fang D., Ran S., Hsiao B.S., Chu B. (2002). Structure and process relationship of electrospun bioabsorbable nanofiber membranes. Polymer.

[48] Clarke E.G.C., Moffat A.C. (1986). Clarke’s Isolation and Identification of Drugs.

[49] Kurowski M. (1988). Zur Pharmakokinetik und Bioverfügbarkeit von Diclofenac-Präparaten nach intramuskulärer Injektion von 75 mg und oraler Gabe von 150 mg Wirkstoff [Pharmacokinetics and biological availability of diclofenac preparations following intramuscular injection of 75 mg and oral administration of 150 mg of active drug]. Z Rheumatol..

[50] Fuoco D. (2015). Cytotoxicity Induced by Tetracyclines via Protein Photooxidation. Adv. Toxicol..

[51] Chen Y., Li H., Wang Z., Tao T., Wei D., Hu C. (2012). Photolysis of Chlortetracycline in aqueous solution: Kinetics, toxicity and products. J. Environ. Sci..

[52] Panzavolta S., Gioffrè M., Focarete M.L., Gualandi C., Foroni L., Bigi A. (2011). Electrospun gelatin nanofibers: Optimization of genipin cross-linking to preserve fiber morphology after exposure to water. Acta Biomaterial..

[53] Dolci L.S., Liguori A., Panzavolta S., Miserocchi A., Passerini N., Gherardi M., Colombo V., Bigi A., Albertini B. (2018). Non-equilibrium atmospheric pressure plasma as innovative method to crosslink and enhance mucoadhesion of econazole-loaded gelatin films for buccal drug delivery. Colloids Surf B Biointerfaces.

[54] Mercatali L., La Manna F., Miserocchi G., Liverani C., De Vita A., Spadazzi C., Bongiovanni A., Recine F., Amadori D., Ghetti M. (2017). Tumor-Stroma Crosstalk in Bone Tissue: The Osteoclastogenic Potential of a Breast Cancer Cell Line in a Co-Culture System and the Role of EGFR Inhibition. Int. J. Mol. Sci..

[55] De Vita A., Recine F., Miserocchi G., Pieri F., Spadazzi C., Cocchi C., Vanni S., Liverani C., Farnedi A., Fabbri F. (2021). The potential role of the extracellular matrix in the activity of trabectedin in UPS and L-sarcoma: Evidences from a patient-derived primary culture case series in tridimensional and zebrafish models. J. Exp. Clin. Cancer Res..

[56] De Vita A., Recine F., Mercatali L., Miserocchi G., Spadazzi C., Liverani C., Bongiovanni A., Pieri F., Casadei R., Riva N. (2017). Primary Culture of Undifferentiated Pleomorphic Sarcoma: Molecular Characterization and Response to Anticancer Agents. Int. J. Mol. Sci..

[57] De Vita A., Miserocchi G., Recine F., Mercatali L., Pieri F., Medri L., Bongiovanni A., Cavaliere D., Liverani C., Spadazzi C. (2016). Activity of Eribulin in a Primary Culture of Well-Differentiated/Dedifferentiated Adipocytic Sarcoma. Molecules.

[58] De Vita A., Liverani C., Molinaro R., Martinez J.O., Hartman K.A., Spadazzi C., Miserocchi G., Taraballi F., Evangelopoulos M., Pieri F. (2021). Lysyl oxidase engineered lipid nanovesicles for the treatment of triple negative breast cancer. Sci Rep..

[59] Miserocchi G., Cocchi C., De Vita A., Liverani C., Spadazzi C., Calpona S., Di Menna G., Bassi M., Meccariello G., De Luca G. (2021). Three-dimensional collagen-based scaffold model to study the microenvironment and drug-resistance mechanisms of oropharyngeal squamous cell carcinomas. Cancer Biol Med..

[60] Mercatali L., Serra P., Miserocchi G., Spadazzi C., Liverani C., De Vita A., Marisi G., Bongiovanni A., Recine F., Pangan A. (2018). Dried blood and serum spots as a useful tool for sample storage to evaluate cancer biomarkers. J. Vis. Exp..

[61] De Vita A., Vanni S., Fausti V., Cocchi C., Recine F., Miserocchi G., Liverani C., Spadazzi C., Bassi M., Gessaroli M. (2021). Deciphering the Genomic Landscape and Pharmacological Profile of Uncommon Entities of Adult Rhabdomyosarcomas. Int. J. Mol. Sci..

