# Supplementary material for: A Modular Composite Device of Poly(Ethylene Oxide)/Poly(Butylene Terephthalate) (PEOT/PBT) Nanofibers and Gelatin as a Dual Drug Delivery System for Local Therapy of Soft Tissue Tumors
Source: Int J Mol Sci. 2022 Mar 17;23(6):3239. doi: 10.3390/ijms23063239 (PMC8948985; doi:10.3390/ijms23063239)
Supplement: Supplementary file 1 [file ijms-23-03239-s001.zip › ijms-1573117-supplementary.pdf]

Supplementary Materials

A modular composite device of poly(ethylene oxide)/poly(butylene terephthalate) (PEOT/PBT) nanofibers and gelatin as dual drug delivery system for local therapy of soft tissue tumors

Anna Liguori*[1], Alessandro De Vita[2], Giulia Rossi[1], Luisa Stella Dolci[3], Silvia Panzavolta[1,4], Chiara Gualandi[1,5], Laura Mercatali[2], Toni Ibrahim[6], Maria Letizia Focarete[1,4]

[1]Department of Chemistry "Giacomo Ciamician" and INSTM UdR of Bologna, University of Bologna, via Selmi 2, 40126, Bologna, Italy; AL: anna.liguori@unibo.it; GR: giulia.rossi49@studio.unibo.it; SP: silvia.panzavolta@unibo.it; CG: c.gualandi@unibo.it; MLF: marialetizia.focarete@unibo.it
Osteoncology Unit, Bioscience Laboratory, IRCCS Istituto Romagnolo Per Lo Studio Dei Tumori (IRST) "Dino Amadori", 47014 Meldola, Italy; ADV: alessandro.devita@irst.emr.it; LM: laura.mercatali@irst.emr.it
[3]Department of Pharmacy and BioTechnology, University of Bologna, Via S. Donato 19/2, 40127 Bologna, Italy; luisastella.dolci2@unibo.it
[4]Health Sciences and Technologies – Interdepartmental Center for Industrial Research (HST-ICIR), Alma Mater Studiorum - Universita ` di Bologna, 40064, Ozzano dell'Emilia, Bologna, Italy; SP: silvia.panzavolta@unibo.it; MLF: marialetizia.focarete@unibo.it
[5]Interdepartmental Center for Industrial Research on Advanced Applications in Mechanical Engineering and Materials Technology, CIRI-MAM, University of Bologna, Viale Risorgimento, 2, 40136, Bologna, Italy; c.gualandi@unibo.it
[6]Osteoncology, Bone and Soft Tissue Sarcomas and Innovative Therapies Unit, IRCCS Istituto Ortopedico Rizzoli, 40136 Bologna, Italy; toni.ibrahim@ior.it

*Correspondence: anna.liguori@unibo.it

Fiber diameter distributions

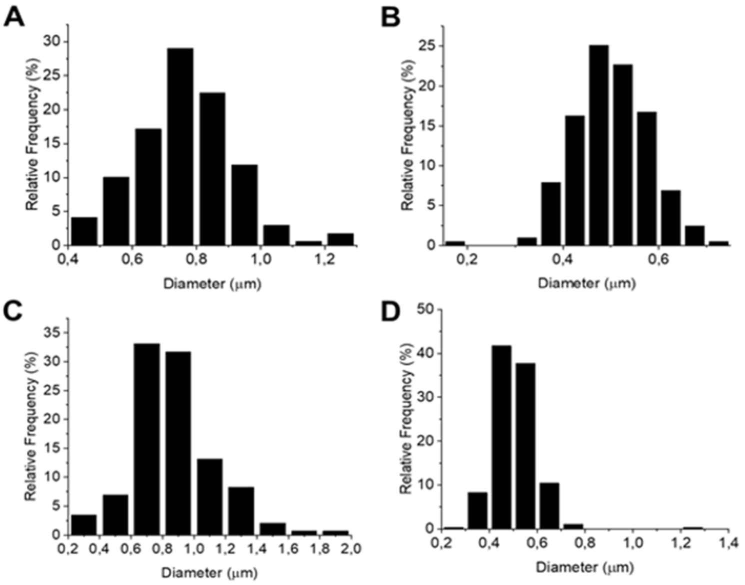

Figure S1. Fiber diameter distributions of 3070 (A), 3070DK (B), 7030 (C) and 7030DK (D).

Thermogravimetric analysis

The results of thermogravimetric analysis are reported in Figure S2 and Table S1. The two copolymers thermally degrade with a similar path, showing: (i) a small weight loss at temperature below 200°C, ascribable to absorbed water, (ii) the main weight loss in the range 360-500°C, followed by (iii) a weak loss at higher temperatures. With the addition of DK in the fibers the amount of absorbed water increases. Moreover, a new weight loss appears before the main one in the range 250-360°C, following the path of DK thermal degradation (black curve).

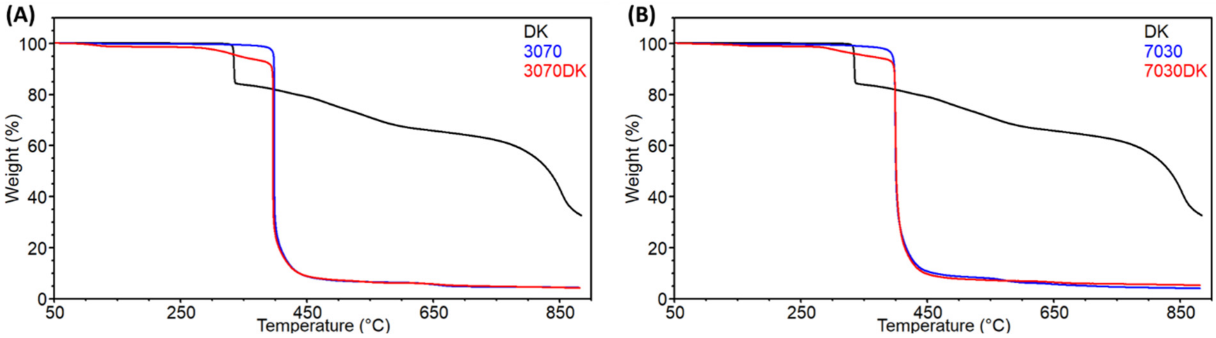

Figure S2. Thermogravimetric analysis of (A) 3070 sample series and (B) 7030 sample series: plain fibers (blue), DK loaded fibers (red) and pure DK (black).

Table S1. Thermogravimetric data of 3070, 3070DK, 7030 and 7030DK electrospun mats.

**Sample**	**T_{max} [°C] RT-250°C**	**Δm [%] RT-250°C**	**T_{max} [°C] 250°C-360°C**	**Δm [%] 250°C-360°C**	**T_{max} [°C] 360°C-500°C**	**Δm [%] 360°C-500°C**	**T_{max} [°C] 500°C-900°C**	**Δm [%] 500°C-900°C**	**m_{res} [%]**
3070	119	0.2	-	-	397	93.3	651	1.9	4.3
3070DK	115	1.4	331	4.5	395	86.6	635	2.5	4.2
7030	125	0.5	-	-	399	90.9	571	4.6	4.0
7030DK	153	1.2	304	3.9	399	87.6	656	2.0	5.2

From the first DSC heating scan (Figure S3 and Table S2), the broad endothermal signals in the range 0°C-70°C and 20°C-120°C for the 3070 and 7030 samples, respectively, highlighted the presence of absorbed water both in the pellets and in the electrospun mats. Moreover, the intensity of the signals turned out to increase in presence of DK, documenting the absorption of higher contents of water in the DK-loaded mats with respect to the plain ones.

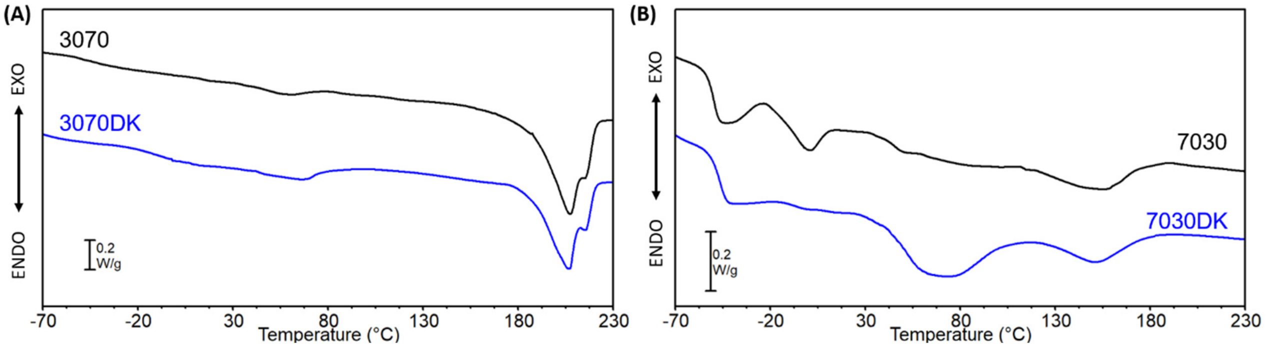

Figure S3. DSC first heating scan of (A) 3070 sample series and (B) 7030 sample series: plain fibers (black), DK loaded fibers (blue).

Table S2. DSC first heating scan. Glass transition temperature (T_g), specific heat capacity (ΔC_p), crystallization temperature (T_c), crystallization enthalpy (ΔH_c), melting temperature (T_m), melting enthalpy (ΔH_m) of 3070, 3070DK, 7030, 7030DK electrospun mats.

**Sample**	**T_g [°C]**	**ΔC_p [J/g·°C]**	**T_c [°C]**	**ΔH_c [J/g]**	**$T_{m,1}$ [°C]**	**$\Delta H_{m,1}$ [J/g]**	**$T_{m,2}$ [°C]**	**$\Delta H_{m,2}$ [J/g]**
3070	-46	0.25	-	-	-	-	207	45
3070DK	n.d.[a)]	n.d.[a)]	-	-	-	-	207	39
7030	-51	0.54	-23	4	0	3	156	9
7030DK	-48	0.52	n.d.[a)]	n.d.[a)]	n.d.[a)]	n.d.[a)]	156	8

[a)] Not detectable, due to the presence of water

Table S3. DSC second heating scan. Glass transition temperature (T_g), specific heat capacity (ΔC_p), crystallization temperature (T_c), crystallization enthalpy (ΔH_c), melting temperature (T_m), melting enthalpy (ΔH_m) of 3070, 3070DK, 7030, 7030DK electrospun mats.

Sample	T_g [°C]	ΔC_p [J/g·°C]	T_c [°C]	ΔH_c [J/g]	$T_{m,1}$ [°C]	$\Delta H_{m,1}$ [J/g]	$T_{m,2}$ [°C]	$\Delta H_{m,2}$ [J/g]
3070	-44	0.23					208	39
3070DK	-37	0.14					205	29
7030	-48	0,39	-23	1	10	18	156	8
7030DK	-43	0,37	-16	1	14	18	153	5

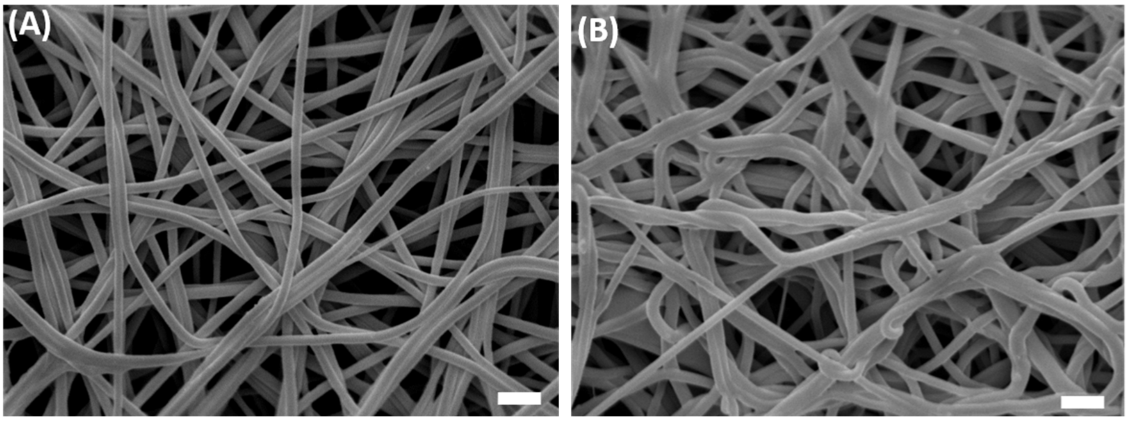

Figure S4. Scanning electron microscopy images of 3070DK (A) and 7030DK (D) after overnight immersion in distilled water. Scale bar: 2 µm
